# The Preparation and Properties of Ti(Nb)-Si-C Coating on the Pre-Oxidized Ferritic Stainless Steel for Solid Oxide Fuel Cell Interconnect

**DOI:** 10.3390/ma17030632

**Published:** 2024-01-28

**Authors:** Xichao Li, Yongchen Chi, Shouli Wei, Xianwei Sun, Jingxiang Zhao, Qiangqiang Hou, Kang Fu, Zuoqiang Dai, Lili Zheng

**Affiliations:** 1 College of Mechanical and Electrical Engineering, Qingdao University, Qingdao 266071, China; lixichao@qdu.edu.cn (X.L.);; 2 National Engineering Research Centre for Intelligent Electrical Vehicle Power System (Qingdao), Qingdao 266071, China; 3 Engineering Technology Center of Power Integration and Energy Storage System, Qingdao University, Qingdao 266071, China

**Keywords:** SOFC interconnect, Ti(Nb)-Si-C coating, oxidation, area specific resistance

## Abstract

Cr_2_O_3_ scale growth and volatilization are the main cause of the performance degradation of solid oxide fuel cells (SOFCs) with an Fe-based ferritic stainless steel (FSS) interconnect. In this work, an amorphous Ti(Nb)-Si-C coating is prepared on the pre-oxidized SUS430 with D.C. magnetron sputtering as the protective coating. The amorphous Ti(Nb)-Si-C coated alloy exhibits significantly enhanced oxidation resistance, and the oxidation kinetics obey the parabolic law with a low parabolic rate of 9.36 × 10^−15^ g^2^·cm^−4^·s^−1^. A dual-layer oxide scale is formed composed of an inner layer rich in Cr_2_O_3_ and an outer layer rich in rutile TiO_2_ and amorphous SiO_2_. MnCr_2_O_4_ appears at the interface between the inner and outer oxide layers. Meanwhile, the amorphous Ti(Nb)-Si-C coating also effectively blocks the outward diffusion of Cr. In addition, the coated steel presents good electrical properties with an area-specific resistance (ASR) of 13.57 mΩ·cm^2^ at 800 °C after oxidation at 800 °C in air for 500 h.

## 1. Introduction

The solid oxide fuel cell (SOFC) is an electrochemical device that can convert chemical energy directly into electricity and heat without combustion or mechanical processes [1,2,3]. It has gained significant attention due to the advantages of high efficiency, fuel flexibility, and low production of pollutants. Due to the low output voltage of 1 V for a single cell, multiple cell stacks should be built up for practical applications. The interconnect becomes an essential multifunctional component in SOFC stacks, which serves as bipolar plates, electrically connecting adjacent cells in series, and acts as the physical separator of fuels on the anode side and air on the cathode side [4,5]. The criteria for interconnect materials are severe, and the materials must meet the demands of high electrical conductivity, be gas-tight, and have an appropriate thermal expansion coefficient (TEC) with adjacent components, good oxidation, carburization resistance, and adequate stability in terms of dimension, microstructure, and phase at operating environments [4,5]. Over the past several decades, LaCrO_3_ and its modified compounds have been widely researched interconnect materials. However, the sintering processes and machining of compact chromite parts are usually difficult, and the manufacturing cost is high. These issues baffle the commercialization of the planar SOFC with LaCrO_3_ interconnects [5]. Recently, the trend in the intermediate temperature operation of SOFCs (600–800 °C) by decreasing the thickness of the ceramic electrolyte makes it feasible to use metallic interconnects [6,7,8]. Ferritic stainless steels (FSSs), such as SUS430 and Crofer22, are currently considered to be the most promising metallic interconnect material due to their high electrical and thermal conductivity, easy manufacture, and low cost [9,10,11]. FSSs possess good oxidation resistance, owing to the formation of Cr_2_O_3_ scales in SOFC environments. However, the formed Cr_2_O_3_ under the service atmosphere can poison the cathode due to the vaporization of Cr species, which may cause the performance degradation of SOFC stacks [12,13]. In addition, the thermally grown Cr_2_O_3_ under the operation condition of SOFC will lead to not only an increase in electrical resistance but also the possibility of coating peeling.

To overcome these problems, one of the most effective modification methods is applying coatings on the FSS surface for thermal protection [14,15]. The requirements for the protective coating include matched TEC with FSS, high electrical conductivity under the operation environment, and chemical stability. The widely studied protective coatings include three categories, which are reactive element oxide (REO) coatings [16,17], rare earth perovskite coatings [18,19], and spinel coatings [20,21,22]. The reactive elements oxides REO coatings can significantly reduce the oxidation rate as well as the area-specific resistance (ASR). However, due to the thin thickness and porous layer structure, REO coatings are considered to be insufficient in inhibiting the outward diffusion of Cr to the oxide scale surface [23,24]. Similarly, the perovskite coatings are ionic conductors, which cannot effectively hinder the diffusion of ions [24,25]. Up to now, spinel oxides, especially (Mn,Co)_3_O_4_, have been considered to be the most suitable protective coatings for FSS interconnects due to their satisfactory electrical conductivity and good oxidation resistance [26,27]. However, the Cr outward diffusion and O inward diffusion must be considered. The long-term stability between the spinel coating and alloy substrate is also a challenge for the spinel coatings [23].

MAX phases are a group of layered ternary compounds with the general formula of M_n+1_AX_n_. M is an early transition metal, A is an IIIA or IVA element, and X is C and/or N. They have gained significant attention due to the merits of both metals and ceramics [28,29]. Ti_3_SiC_2_, one of the most typical MAXs, presents unique properties, such as low density, high modulus, and fracture toughness. At the same time, it meets the requirements for SOFC interconnect, such as high thermal and electrical conductivity, good resistance to oxidation, and machinability. Meanwhile, its thermal expansion coefficient TEC (~9.2 × 10^−6^ K^−1^ (20–1000 °C)) matches with the yttria-stabilized zirconia (YSZ, 10.5 × 10^−6^ K^−1^) of the electrolyte for SOFC [30,31], which can avoid the mismatch of TEC with adjacent components in SOFC stacks. Our previous works [31,32,33,34,35] presented that Nb-, W-, or Ta-doped (Ti,M)_3_SiC_2_ (M = Nb, W, or Ta) possess excellent oxidation resistance and a low ASR after oxidation (500~700 h) under the simulated SOFC cathode atmosphere. The oxide scales formed on the surface are composed of a mixture of M-doped rutile-TiO_2_ and amorphous-SiO_2_. The doping can decrease the concentrations of both oxygen vacancies and titanium interstitials in r-TiO_2_. The decreased concentrations of both oxygen vacancies and titanium interstitials can not only improve the oxidation resistance of the solid solution but can also increase the electrical conductivity of TiO_2_ by increasing the concentration of electrons. Among these solid solutions, (Ti_0.95_Nb_0.05_)_3_SiC_2_ possesses the best performance, which endows it with great potential as an interconnect for intermediate-temperature solid oxide fuel cells (IT-SOFCs) [32]. Previous works indicated that MAX phase coatings can be prepared with the D.C. magnetron sputtering method, inheriting the excellent properties of bulk materials [36]. Therefore, (Ti,Nb)_3_SiC_2_ coatings may make full use of their high oxidation resistance and high electrical properties as interconnect protective coatings.

The inter-diffusion between coating and substrate is a critical problem. For example, Cr in the substrate may diffuse into a spinel coating, resulting in decreased electrical conductivity of spinel and eventually peeling of coating in long-term operation [37]. Many works indicate that pre-oxidation can effectively inhibit the inter-diffusion between coating and alloy, resulting in the increased stability of coatings on alloys. Ardigo-Besnard et al. [38] reported that the growth of iron oxides on AISI 441 can be effectively avoided by pre-oxidation by hindering the outward diffusion of Fe. The electrical conductivity of the formed oxide layer is also improved. Amendola et al. [39] studied the effect of pre-oxidation on the oxidation of Crofer 22APU. It was found that pre-oxidation is effective in inhibiting iron outward diffusion, which can reduce the growth rate of the oxide scale during the 200 h oxidation in air. Talic et al. [40] investigated the effect of pre-oxidation conditions (in air, H_2_, and N_2_-1% H_2_-9% H_2_O atmosphere) on the oxidation behavior of Crofer 22APU at 800 °C in air. It was deduced that the pre-oxidation treatment in N_2_-9%H_2_-1%H_2_O for 5 h at 1100 °C could decrease the oxidation kinetics of the alloy.

In this work, a (Ti,Nb)-Si-C coating is fabricated on a pre-oxidized SUS430 substrate with D.C. magnetron sputtering. The pre-oxidation is conducted at 800 °C in air for 10 h in a tube furnace. The phase composition of the coating is identified, and the cross-section and surface morphologies are examined. The oxidation behaviors and electrical properties of the coated pre-oxidized SUS430 are systematically investigated at 800 °C in air atmosphere. The barrier effect of the (Ti,Nb)-Si-C coating on the Cr outward diffusion behavior is discussed.

## 2. Experimental Procedure

### 2.1. Material Preparation

**Figure 1:**
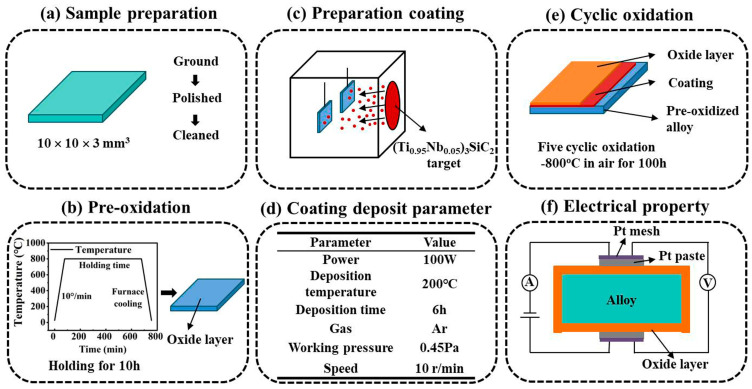
Sample preparation and research process diagram.

The sample preparation and research methods are depicted in Figure 1. The alloy substrate is commercially available SUS430 stainless steel. The SUS430 substrate was cut into pieces with a size of 10 mm × 10 mm × 3 mm using electric discharge machining (EDM). The samples were ground down to 2000 grit SiC paper, polished with 1 µm diamond paste, ultrasonic cleaned in acetone, and then washed with ethanol and deionized water (Figure 1a). Afterward, the samples were pre-oxidized at 800 °C in air for 10 h in a tube furnace (Figure 1b). The (Ti_0.95_Nb_0.05_)_3_SiC_2_ bulk material was used as a target with dimensions of *Φ*60 mm × 6 mm. (Ti_0.95_Nb_0.05_)_3_SiC_2_ bulk was fabricated with the in situ reaction/hot pressing method, as has been described elsewhere [31,32].

The coating was prepared using a JP−500BY magnetron sputtering system (Liaoning Beiyu Vacuum Technology Co., Ltd., Tieling, China) in D.C. mode (Figure 1c). The distance between the target and substrate was 10 cm. The substrate holder rotated at 10 rpm during the sputtering process to obtain the homogeneous coating. Before sputtering, the chamber was pumped down to a base pressure of 5 × 10^−4^ Pa to eliminate the oxygen left in the chamber. Then, the chamber was heated to the setting temperature of 200 °C. The inert Ar gas (99.999%) was introduced into the sputtering chamber as the working gas. Prior to deposition, Ar^+^ ions were used to remove the surface contaminants on the target for 15 min by applying a negative bias of 500 V in 1 Pa Ar. Afterward, the deposition was started with the working pressure of Ar kept at about 0.45 Pa by fine-tuning the gas flow valve. The applied power on the target was selected to be 100 W. No bias voltage was applied on the sample holder (Figure 1d) during deposition. After deposition for 6 h, the samples were taken out for the research tests.

### 2.2. Research Methods

#### 2.2.1. Oxidation Tests

The cyclic oxidation test of the coated and uncoated samples was conducted in a tube furnace at 800 °C in air for 500 h. During the whole oxidation period, after every 100 h oxidation, the samples were cooled down with furnace cooling to room temperature in air. Then, the samples were weighed using a micro-balance with the accuracy of 1 × 10^−5^ g. After weighting, the samples are put back into the hot furnace again for the next 100 h oxidation at 800 °C in air (Figure 1e).

#### 2.2.2. ASR Measurement

The ASR of the oxidized samples was determined with the 2-probe 4-point method at 600~800 °C in air. A stable current of 10 mA was supplied by a Precision Programmable Current Source (YL4012), and the voltage drop was recorded with a FLUKE 8845A 6-1/2 Digit Programmable Multimeter. The electrical resistance of the surface can be obtained using Ohm’s low:

(1) ASR=U/2I·S

where *ASR* is the area-specific resistance, *I* is the applied current, *U* is the corresponding voltage, and *S* is the tested surface area (Figure 1f).

#### 2.2.3. Characterization Methods

The phase composition of the coating and the oxide scale products were identified with X-ray diffraction in a D/max-2500PC diffractometer (Rigaku, Tokyo, Japan). The surface and cross-section morphology of the coating and the oxide scale were observed with a field-emission scanning electron microscope (FESEM, JEOL JSM- 7800F, JEOL Ltd., Tokyo, Japan) equipped with an energy-dispersive X-ray spectrometer (EDS) for chemical composition analysis. A secondary electron mode was used for surface morphology, and a back-scattered electron mode was used for cross-section observation. The chemical composition of the coating and oxide scale was also determined with X-ray photoelectron spectroscopy (XPS). XPS measurements were performed on the sample surfaces using a surface analysis system Thermo Scientific K-Alpha (Thermo Scientific, ESCALAB 250Xi, Thermo Fisher, MA, USA) equipped with Al radiation (Kα = 1486.6 eV).

## 3. Results and Discussion

### 3.1. Characterization of the Coating

**Figure 2:**
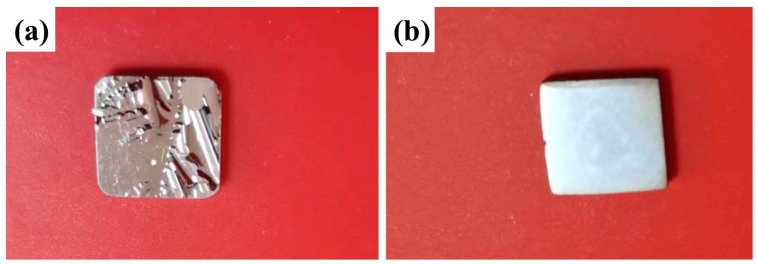
Optical photographs of the coatings on the surface of the (**a**) polished and (**b**) pre-oxidized alloys.

Figure 2a,b shows the optical photographs of coatings sputtered on the surface of the polished and pre-oxidized alloys, respectively. It can be clearly seen that the coating deposited on the surface of the polished sample peels off from the substrate, implying that the smooth surface is adverse to the deposition of Ti-Si-C film. Differently, the coating deposited on the surface of the pre-oxidized sample appears macroscopically flat, integrated, and adhered to the substrate. It is indicated that the thin oxide scale formed on the surface of the pre-oxidized sample can provide the nucleation site for the coating, which promotes the nucleation and growth of the coating and improves adhesion [41].

**Figure 3:**
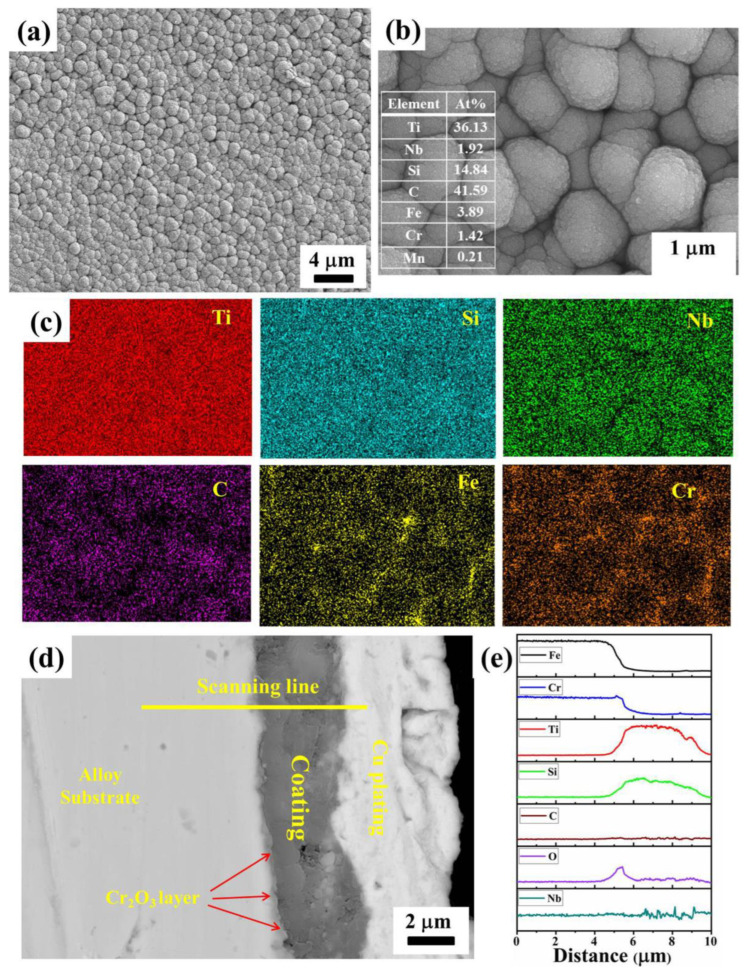
(**a**,**b**) Surface and (**c**) element mapping profiles on the coated sample; (**d**) cross-sectional image of the deposited coating; and (**e**) EDS line-scanning along the line in (**d**).

Figure 3a,b shows the surface microscopies of the deposited coating on the pre-oxidation sample. It can be seen that the coating is flat and composed of uniformly close-packed particles. The element mappings are depicted in Figure 3c. The quantitative results of EDS are inserted in Figure 3b. It can be deduced that the particles mainly contain the elements Ti, Si, and C. The elements from the alloy substrate can be detected through the gap of the particles. In addition, Nb is also detected in the deposited coating with a doping concentration similar to the target material. The corresponding cross-sectional morphology in Figure 3d indicates that the coating is dense, integrated, and homogeneous with a thickness of about 3.6 μm. Moreover, the coating is tightly bonded to the substrate. Figure 3e shows the corresponding EDS line scanning profiles along the yellow line in Figure 3d. Clearly, Ti and Si elements are uniformly distributed in the coating. Due to the low concentration, the EDS line scanning profile of Nb is not obvious. There is a very thin layer between the substrate and the coating, which is marked by a red arrow. Combining the results of EDS line-scanning, the inner layer is deduced to be the Cr_2_O_3_ layer, which was formed during the pre-oxidation period.

**Figure 4:**
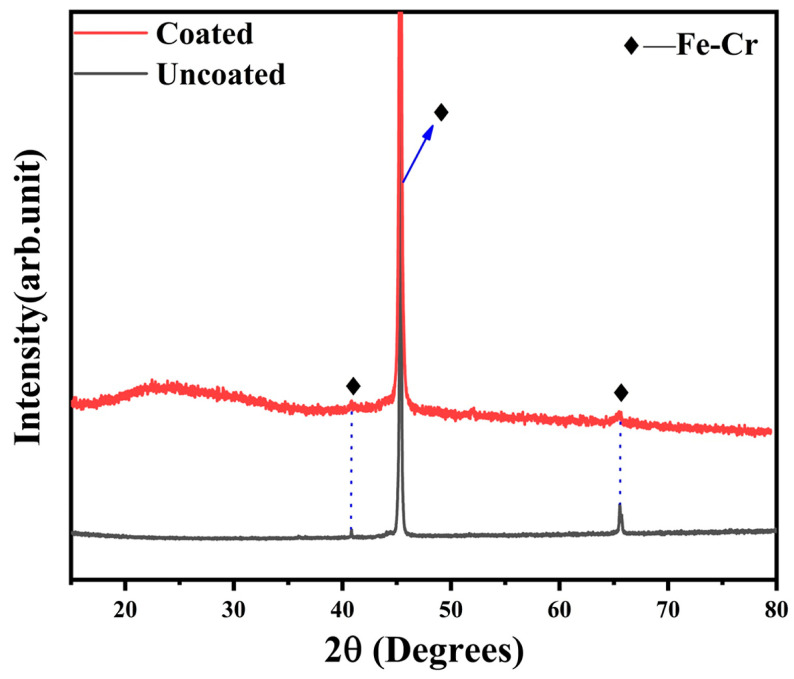
XRD patterns of the coated and uncoated pre-oxidized alloys.

Figure 4 presents the XRD patterns of the coated and uncoated pre-oxidized alloys. Compared to the bare substrate, no other peak is detected on the coated sample except an amorphous bulge between 20 and 30°. It is suggested that the deposited coating is an amorphous Ti(Nb)-Si-C coating. The amorphous coating is caused by over-cooling during the sputtering process [42].

### 3.2. Oxidation Behavior

**Figure 5:**
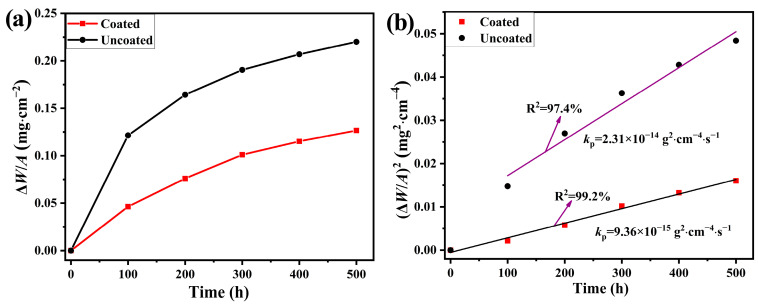
(**a**) Mass gain and (**b**) oxidation kinetics of the coated and uncoated samples at 800 °C in air for 500 h.

Figure 5a shows the oxidation kinetics (mass gain per unit surface area vs. oxidation time) of the coated and uncoated samples during the cyclic oxidation at 800 °C in air atmosphere up to 500 h. It can be seen that the mass gain in the two samples increased steadily during the whole oxidation period, indicating no oxide scale peeling. Furthermore, the coated sample exhibits less mass gain during the whole oxidation period. As shown in Figure 5b, the square of the mass gain per unit surface area of the coated sample is approximately linear (with the relative reference of R^2^ being 99.2%) as the function of time in the whole period, which verified that the oxidation kinetics follow the parabolic law. That is to say, ion diffusion through the formed oxide layer is the controlled step for the oxidation of the coated sample. For the pre-oxidized alloy, the mass gains increase tardily with time after the first 100 h, indicating that the protective Cr_2_O_3_ layer was formed therein. The square of the mass gain per unit surface area of the pre-oxidized alloy vs. oxidation time also approximately follows the linear law (with the relative reference of R^2^ being 97.4%). Based on Wagner’s oxidation theory [43], while the curve is parabolic, the oxidation rate constant is satisfied by the following equation:

(2) $${\left(\Delta W/A\right)}^{2}=k_{p}t$$

where Δ*W* is the mass gain, *A* is the total surface area exposed to the oxidizing atmosphere, *k*_p_ is the parabolic rate constant, and *t* is the oxidation time. By fitting the curves in Figure 5b, the corresponding *k*_p_ are obtained, which are also added in Figure 5b. In addition, the *k*_p_ determined from this work and other references are listed in Table 1. It can be seen that the *k*_p_ for the coated sample is about 40.5% of that for the pre-oxidized alloy, indicating that the Ti(Nb)-Si-C amorphous coatings are beneficial to the enhanced oxidation resistance of SUS430. By comparison with the Mn-Co coating, it is found that the Ti(Nb)-Si-C amorphous coatings possess a relatively high oxidation resistance under the simulated cathode atmosphere of SOFC.

**Figure 6:**
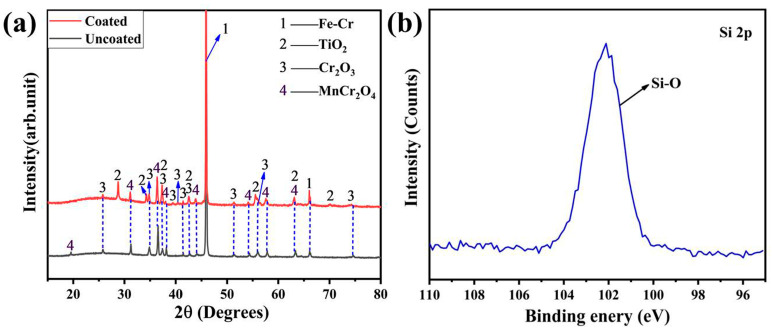
(**a**) XRD patterns of the oxide on the coated and uncoated samples after oxidation at 800 °C in air for 500 h and (**b**) the XPS spectrum of Si 2p.

The XRD patterns of the samples after oxidation for 500 h are depicted in Figure 6a. After oxidation for 500 h, the oxide scales formed on the uncoated alloy are composed of Cr_2_O_3_ [40] and MnCr_2_O_4_ [1]. Compared with the uncoated sample, the TiO_2_ [33] phase is dominated by the oxide scale of the coated sample. Figure 6b shows the XPS spectrum of Si 2p. The Si-based oxide phase is determined to be silicon species since the binding energy of Si 2p is 102.3 eV [45]. It is deduced that amorphous SiO_2_ is formed in the oxide layer of the coated sample, which is the same as the case of (Ti,Nb)_3_SiC_2_ bulk [31,32].

**Figure 7:**
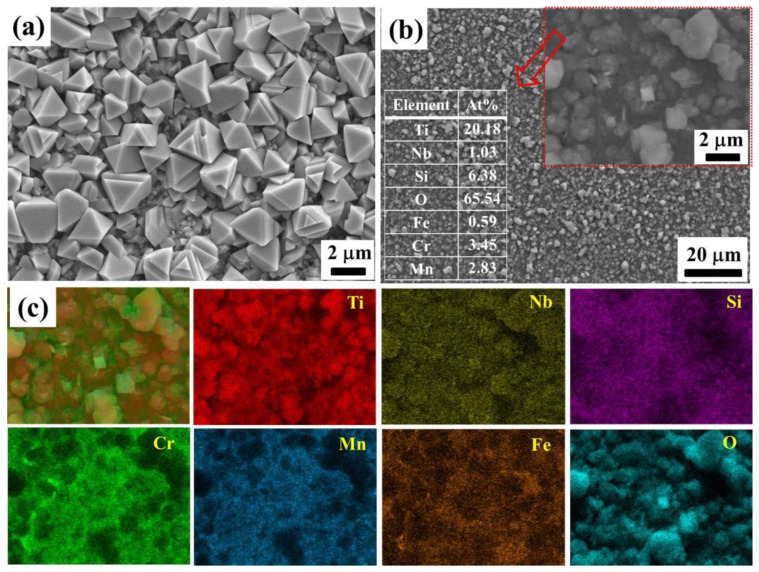
Surface morphology of the (**a**) coated and (**b**) uncoated samples after oxidation at 800 °C in air for 500 h, and (**c**) element mapping profiles on the coated sample.

Figure 7 presents the surface morphology and element mappings of the samples after oxidation at 800 °C in air for 500 h. From Figure 7a, it can be seen that the surface of the pre-oxidized sample is covered with polyhedral grains, which are loosely packed. The analogous morphology of the oxide scale is also found in a previous reference [46]. Figure 7b shows the morphology of the oxide scale formed on the surface of the coated sample. It is relatively flat without cracks and spalling, indicating good adhesion between the oxide scales and the substrate. The inset in Figure 7b shows that the oxide scale is mainly composed of particles and colloidal substances. Combined with the XRD and the element mapping results in Figure 7c, it is deduced that the particles on the oxide surface are mainly rutile TiO_2_. The colloidal substance is amorphous SiO_2_ in the oxide layer. Furthermore, it is found that the distribution of the Nb map is almost the same as Ti, which suggests that Nb was also doped into TiO_2_ after oxidation. The area EDS mapping profiles are inserted in Figure 7b. The results reveal that the atomic ratio of Nb:Ti is about 4.86:95.14, which is similar to that of the (Ti,Nb)_3_SiC_2_ target. This phenomenon is the same as the case of the oxidation of (Ti,Nb)_3_SiC_2_ bulk [31,32]. The elements Cr, Mn, and Fe are also detected on the surface of the coated sample, and the distribution maps are similar.

**Figure 8:**
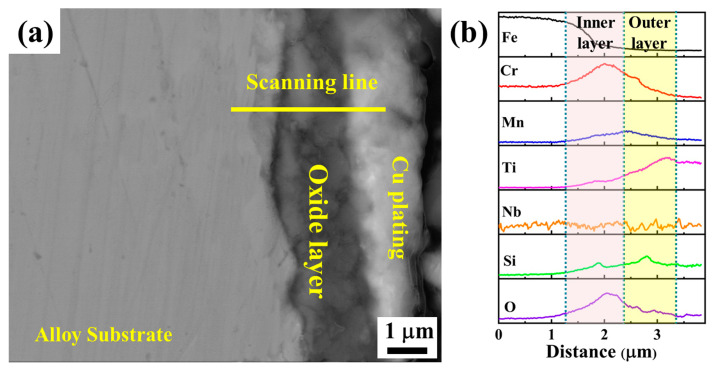
(**a**) Cross-section morphology and (**b**) EDS line scanning along the yellow line in (**a**) of the coated sample after oxidation at 800 °C in air for 500 h.

Figure 8 presents the cross-section morphology of the coated sample after being oxidized at 800 °C in air for 500 h and the corresponding EDS line scanning along the yellow line. It is revealed that the oxide layers formed on the surface exhibit good adhesion to the substrate, and no voids or spallation are found at the interface of the oxide scale and substrate after five thermal cycles. The oxide scale has a double-layer structure. Combining the XRD and the EDS results, it is concluded that the inner layer is rich in Cr_2_O_3_ with a little MnCr_2_O_4_, TiO_2_, and SiO_2_. Meanwhile, the outer layer is mainly composed of TiO_2_ and SiO_2_. At the interface of the inner and outer layers, the Mn content increases, suggesting the formation of MnCr_2_O_4_ by the outward diffusion of Mn and reacting with Cr_2_O_3_. It is worth noting that the content of Cr decreases obviously in the outer layer, suggesting that the Ti(Nb)-Si-C coating can effectively inhibit the outward diffusion of Cr during oxidation, which can further prevent Cr poisoning to the cathode. Meanwhile, the outward diffusion of Mn and Fe atoms is also restrained. Furthermore, it is found that although no crystalline (Ti,Nb)_3_SiC_2_ phase is obtained in the coating, the amorphous Ti(Nb)-Si-C coating still exhibits the excellent oxidation resistance of the (Ti,Nb)_3_SiC_2_ bulk material.

### 3.3. Electrical Property

**Figure 9:**
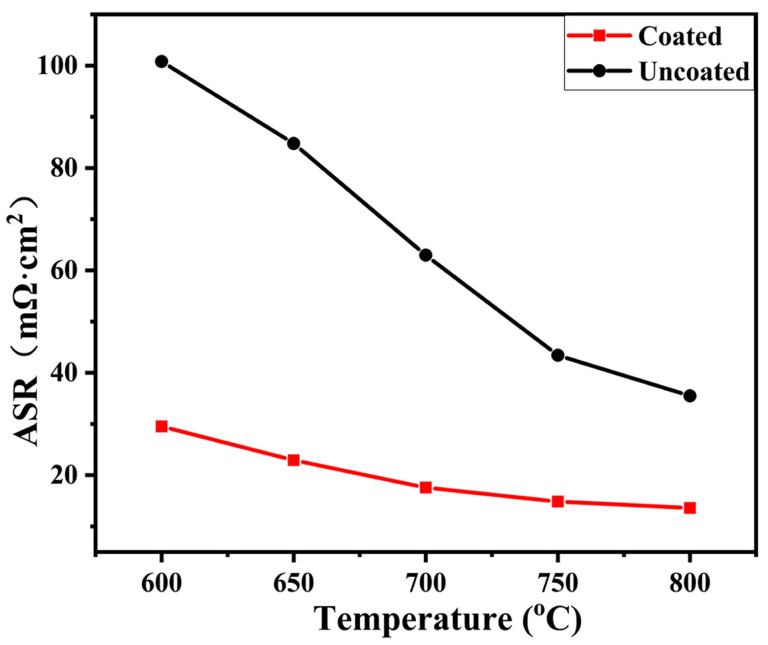
The ASR of the coated and uncoated pre-oxidized samples after oxidation at 800 °C in air for 500 h.

A low and stable electrical resistance of interconnect under SOFC operation conditions is beneficial for the high voltage output of the SOFC stack. Because the electrical resistance of the alloy substrate is much lower than the oxide scale under SOFC working atmosphere, the electrical resistance of the interconnect mainly comes from the oxide scale formed on the alloy under servicing conditions. ASR is normally chosen to assess the electrical properties of the oxide scales formed on an interconnect, which both reflects the electrical conductivity of the scale and the thickness of the oxide layer. Generally, the upper limit ASR of an interconnect scale is 100 mΩ·cm^2^ after 40,000 h service [47]. Figure 9 presents the ASR vs. temperature for the coated and uncoated samples after oxidation at 800 °C in air for 500 h. Apparently, the ASR values decrease with increasing temperature for the two samples, which is consistent with the properties of the semiconductor. It is also proved that the electrical resistance of the oxide scale plays a major role in the ASR. The ASR for the coated sample is lower than that of the uncoated one in the temperature range of 600~800 °C. The ASR for the Ti(Nb)-Si-C coated and uncoated samples at 800 °C are 13.57 and 35.49 mΩ·cm^2^, respectively. In addition, the values of ASR determined from this work and in other references are listed in Table 2. When the oxide scale thermally grows, the ASR value is generally proportional to the thickness of the oxide scale. That means the ASR is proportional to $\sqrt{k_{p}\cdot t}$. With comparison, it is found that the Ti(Nb)-Si-C coated sample possesses relatively high electrical conductivity after oxidation. The electrical conductivity of TiO_2_, SiO_2_, and Cr_2_O_3_ is 1 × 10^2^ Ω·cm at 900 °C, 7 × 10^6^ Ω·cm at 600 °C, and 1 × 10^2^ Ω·cm at 800 °C, respectively. That is to say, the electrical conductivity of the mixture oxide scale of TiO_2_ and SiO_2_ should be lower than that of the Cr_2_O_3_ oxide layer. The reason for this contradiction is the Nb doping in the rutile TiO_2_, which can increase the electrical conductivity of rutile TiO_2_ [31,32,33,34,35]. Moreover, the thickness of the oxide scale on the coated sample is much thinner than that of the uncoated sample. Therefore, the coated sample possesses a lower ASR than that of the uncoated sample.

## 4. Conclusions

An amorphous Ti(Nb)-Si-C coating is prepared on a pre-oxidized SUS430 alloy with D.C. magnetron sputtering technology. The coated samples exhibit high oxidation resistance and electrical properties under the simulated cathode atmosphere of SOFCs. The following conclusions are drawn:

(1) The amorphous Ti(Nb)-Si-C coating is dense, flat, and tightly bonded on the pre-oxidized SUS430 substrate. The Ti and Si elements are uniformly distributed in the coating.
(2) The amorphous Ti(Nb)-Si-C-coated alloy exhibits good oxidation resistance (*k*_p_ = 9.36 × 10^−15^ g^2^·cm^−4^·s^−1^), with the oxidation following the parabolic law. The oxide scale is double-layer structured with the inner layer being rich in Cr_2_O_3_ and the outer layer being rich in rutile TiO_2_ and amorphous SiO_2_. MnCr_2_O_4_ appears at the interface of the inner and outer layers. The amorphous Ti(Nb)-Si-C coating effectively blocks Cr outward diffusion.
(3) The amorphous Ti(Nb)-Si-C coating presents good electrical properties with a low ASR of 13.57 mΩ·cm^2^ at 800 °C after oxidation at 800 °C in air for 500 h.

## Figures and Tables

**Table 1:** Kp determined from this work and other references.

Steel	Coating Type	Testing Condition	Oxidation Rate Constant (g^2^·cm^−4^·s^−1^)	Reference
Sus430	Uncoated	800 °C in air	9.36 × 10^−15^	This work
Sus430	Ti(Nb)-Si-C	800 °C in air	2.31 × 10^−14^	This work
Sus430	Uncoated	800 °C in air	8.5 × 10^−14^	[44]
Sus430	Uncoated	800 °C in air	4.78 × 10^−14^	[1]
Sus430	Mn-Co	800 °C in air	1.22 × 10^−14^	[1]

**Table 2:** ASR determined from this work and other references.

Steel	Coating Type	Testing Condition	ASR (mΩ·cm^2^)	Reference
Sus430	Uncoated	800 °C in air for 500 h	35.49	This work
Sus430	Ti(Nb)-Si-C	800 °C in air for 500 h	13.57	This work
Sus430	Uncoated	800 °C in air for 100 h	20	[44]
Sus430	Uncoated	800 °C in air for 1250 h	27.2	[1]
Sus430	Mn-Co	800 °C in air for 1250 h	28.6	[1]

## Data Availability

The data presented in this study are available upon request from the corresponding author.
